# Idiopathic Basal Ganglia Calcification: Fahr’s Syndrome, a Rare Disorder

**DOI:** 10.7759/cureus.5895

**Published:** 2019-10-12

**Authors:** Ranjani Thillaigovindan, Eswaran Arumugam, Rathika Rai, Prabhu R, R Kesavan

**Affiliations:** 1 Prosthodontics, Thai Moogambigai Dental College and Hospital, Chennai, IND; 2 Prosthodontics, Thai Moogambigai Dental College & Hospital, Chennai, IND; 3 Public Health Dentistry, Thai Moogambigai Dental College & Hospital, Chennai, IND

**Keywords:** basal ganglia calcification, fahr’s syndrome, neurological disorder, idiopathic, missing tooth

## Abstract

Fahr’s syndrome is a rare neurological disorder with varied clinical manifestations. It is characterized by the progressive deposition of calcium in the walls of the blood vessels of basal ganglia and dentate nuclei of the cerebellum in young and middle-aged people. It is important for neurologists, geneticists, psychiatrists, dentists, and other appropriate care specialists to have a thorough knowledge of this syndrome as any of them could be the first person to diagnose the disease. This case report of Fahr’s syndrome presents the signs and symptoms of the patient and the treatment for oral conditions were done.

## Introduction

Fahr’s disease or Fahr’s syndrome is a rare neurological disorder, which is most commonly transmitted as an autosomal dominant trait. It may also occur sporadically. It is characterized by the abnormal deposition of calcium in areas of the brain that control movement. It includes the basal ganglia, thalamus, dentate nucleus, cerebral cortex, cerebellum, and hippocampus. These deposits consist of minerals like calcium phosphate and calcium carbonate. They also consist of metals like iron, copper, magnesium, silver, and cobalt. Most calcification occurs bilaterally and symmetrically. Rarely, unilateral deposits also occur [1].

It typically affects individuals in their third and fourth decades although childhood cases are also reported. It is also known as idiopathic basal ganglia calcification, striopallidodentate calcification, and calcinosis nucleorum [1-2].

There are many neurological and neuropsychiatric symptoms and movement disorders associated with Fahr’s syndrome. Certain diagnostic criteria are derived from Moskowitz et al. (1971), Ellie et al. (1989), Manyam (2005), such as bilateral calcification of the basal ganglia visualized on neuroimaging, progressive neurologic dysfunction, absence of biochemical abnormalities, infectious, toxic, or traumatic cause, and family history consistent with autosomal dominant inheritance [3-5].

Oral manifestations include dental dysplasia, delayed tooth eruption, and impacted supernumerary teeth. Impacted teeth should be removed cautiously. The rehabilitation of function, speech, and aesthetics can be done. Treatment strategies mainly consist of symptomatic relief like the prescription of anti-epileptics for seizures, antipsychotics for depression, and clonazepam for dystonia. With little evidence in the literature, it can be suggested that early diagnosis and treatment can reverse the calcification process so that there can be a complete recovery from neurologic disorders.

## Case presentation

**Figure 1:**
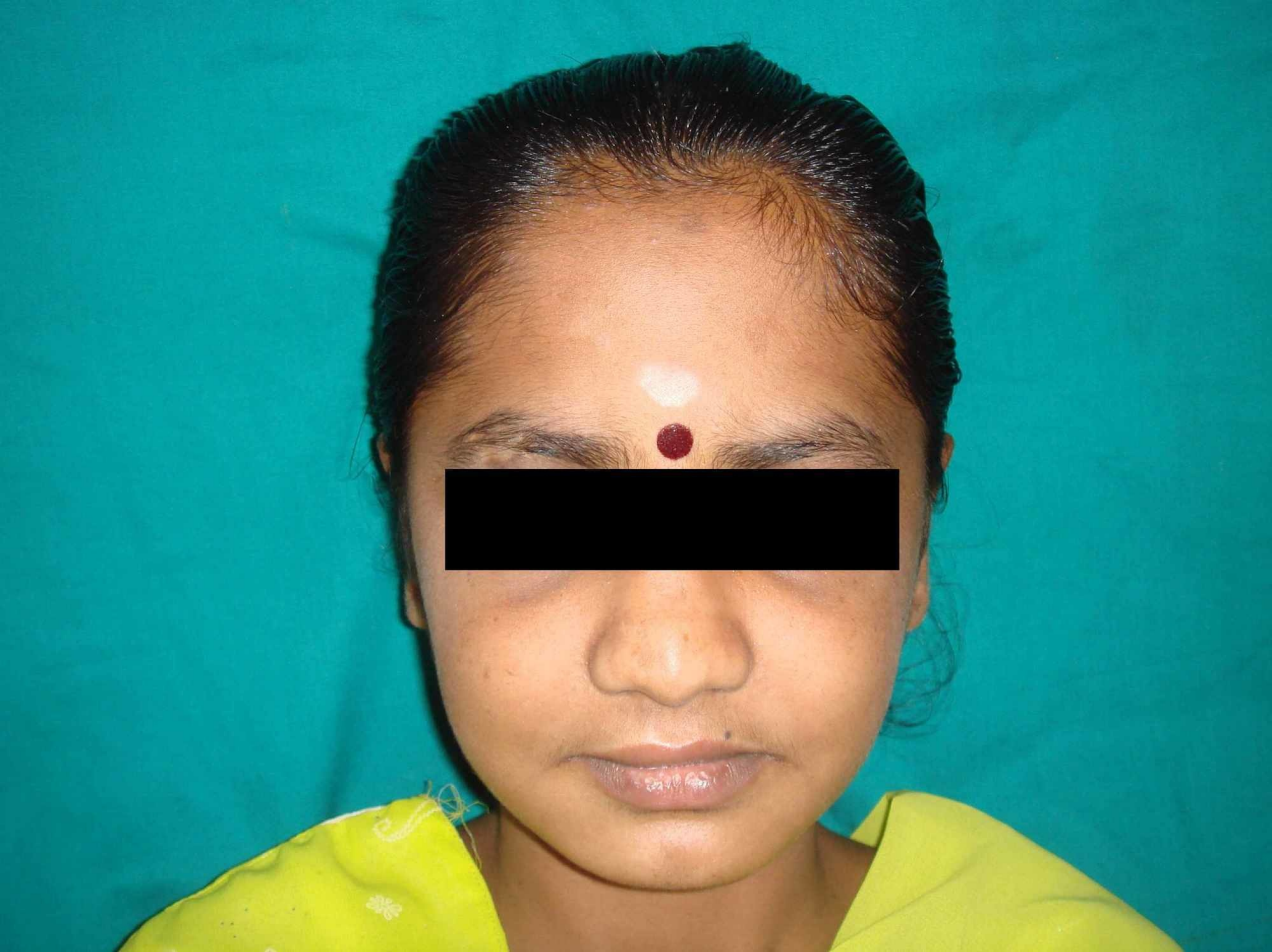
Frontal view of the patient

**Figure 2:**
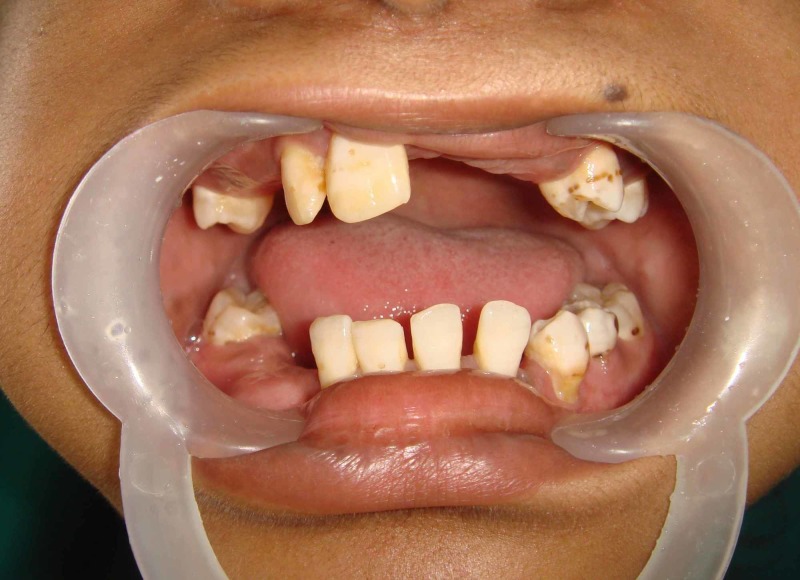
Intra-oral view

A 15-year-old female patient came to the ACS Medical College outpatient department with mental health disorders. She was later referred to Thai Moogambigai Dental College and Hospital for the rehabilitation of missing teeth (Figure 1). She wanted to replace her missing teeth. On taking a history, it was found that a few teeth failed to erupt due to delayed tooth eruption, as shown in Figure 2. She had difficulty in eating and speaking. She also presented with a history of progressive deterioration of mental health and mild deterioration of motor functions for the past two years. Slow or slurred speech and dysarthria were present. Her father also had a family history of mental illness and movement disorder for which he is under treatment.

**Figure 3:**
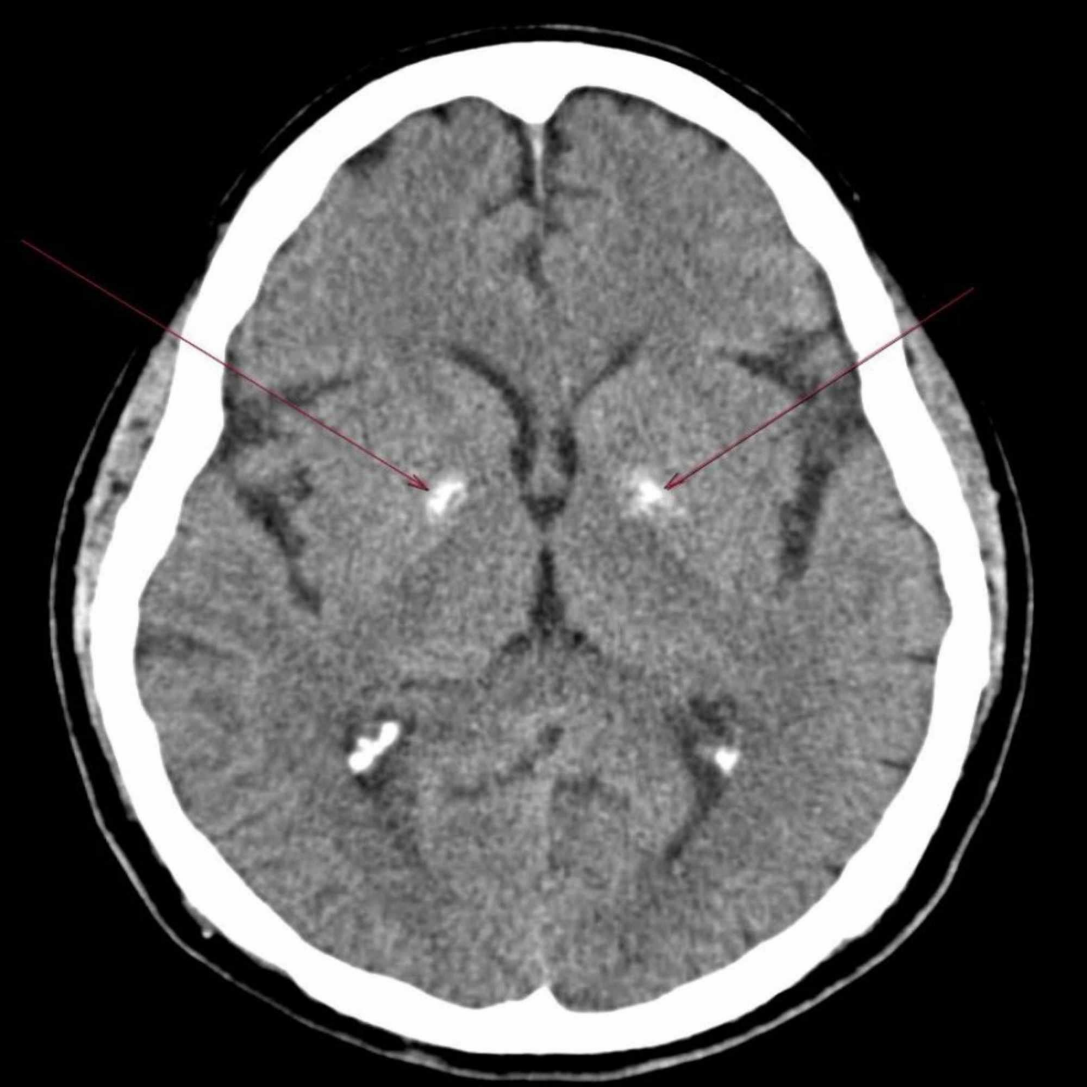
Computerized tomography of the brain

Blood investigations showed normal blood glucose, serum calcium, phosphorus, and parathyroid hormone levels. There was no evidence of any infectious disease of the eye. Computed tomography (CT) of the brain showed bilateral symmetrical areas of calcification in the basal ganglia, as seen in Figure 3.

The pathophysiology of this syndrome is the development of calcification within the vessel wall and in the perivascular space, ultimately extending to the neuron. This results in damage to the neurons and thus initiation of calcification. Progressive basal ganglia calcification compresses the vessel lumen, initiating the cycle of reduced blood flow, neural tissue injury, and mineral deposition. Deposits are composed of calcium phosphate, mucopolysaccharides, and metals, including iron, copper, silver, and cobalt.

**Figure 4:**
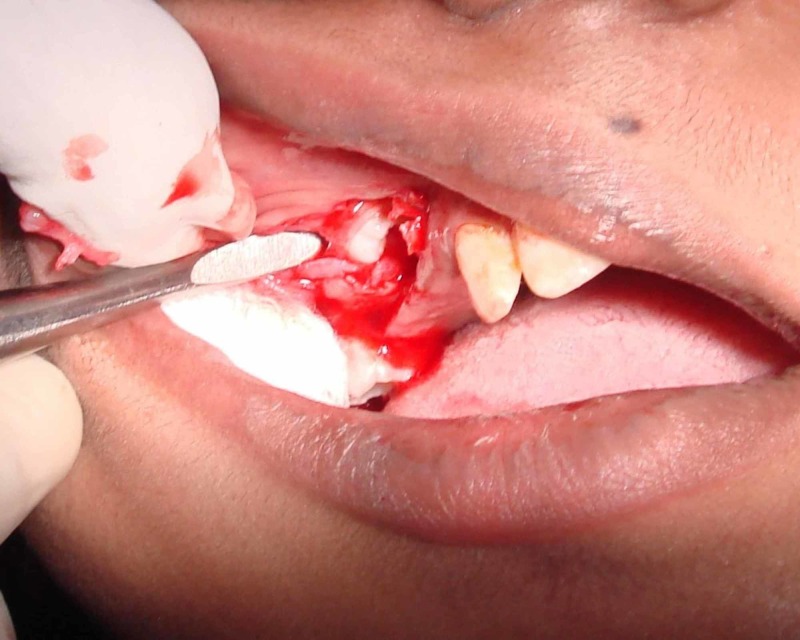
Extraction of unerupted teeth in the maxilla.

**Figure 5:**
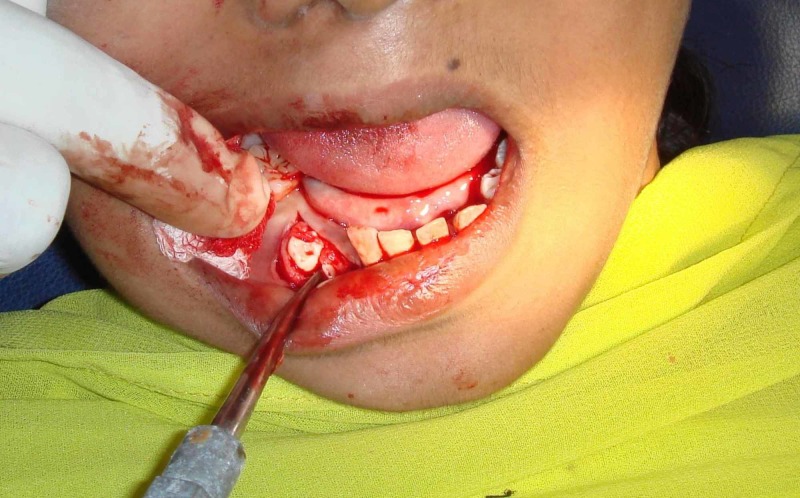
Extraction of unerupted teeth in the mandible

**Figure 6:**
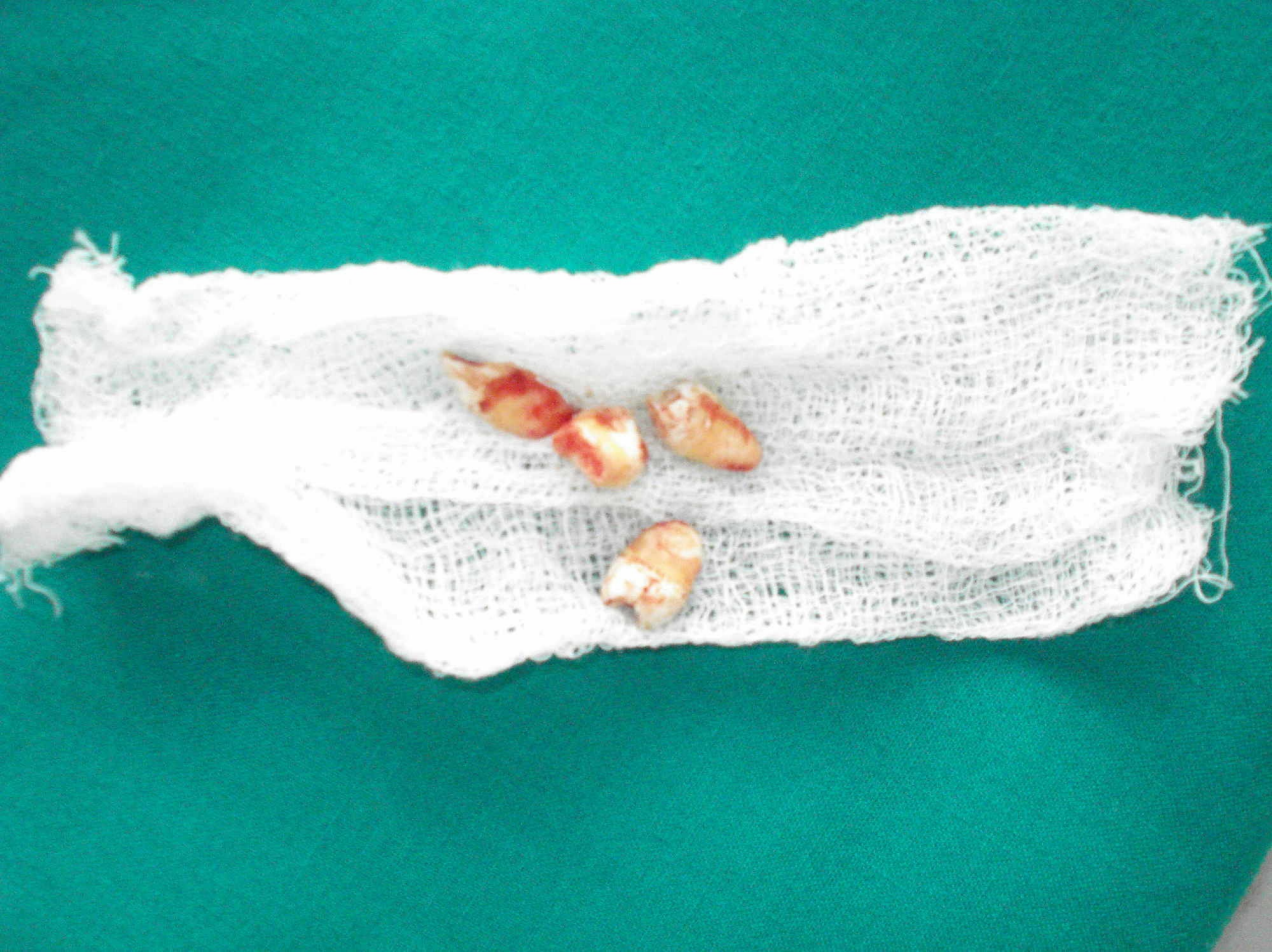
Extracted teeth displayed on gauze

**Figure 7:**
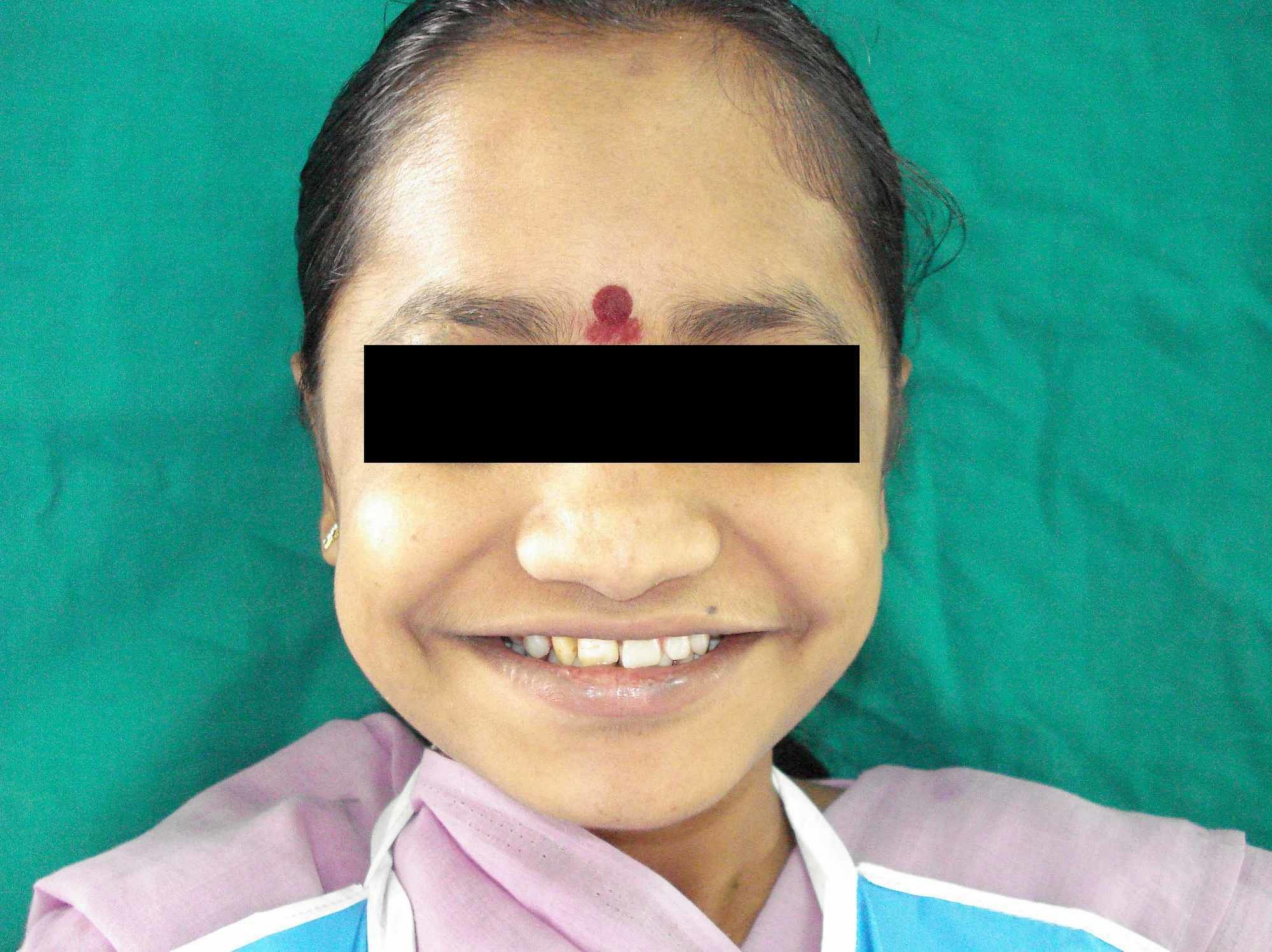
Patient rehabilitated with a partial denture

On the basis of history, clinical features, CT findings, and normal blood investigations, a diagnosis of Fahr’s syndrome was made. As the treatment is based on symptomatic relief, the patient’s behavioral problems were treated with antipsychotics. Oral problems were treated with the extraction of unerupted impacted teeth, as seen in Figures 4-6. Later, the missing teeth were rehabilitated with a temporary removable prosthesis, as shown in Figure 7. The patient will be recalled after three months of complete bone formation for a fixed prosthesis.

Patient consent was obtained to use her medical history and photographs for study purposes.

## Discussion

Fahr’s syndrome is basically idiopathic basal ganglia calcification, which is not affected by age. The calcium deposits usually form within the vessel wall and in the perivascular space and finally extend to the neuron. The pathogenesis of this syndrome is believed to be due to defective iron transport and free radical production, resulting in tissue damage. This leads to the initiation of brain calcification. This calcification compresses the vessel lumen, initiating a cycle of impaired blood flow and injury to neurons. Most calcifications occur bilaterally and symmetrically while a few occur unilaterally. Abnormal metabolism of calcium and phosphorus is noted in some patients [6-7].

Although the etiology of this syndrome does not pinpoint a specific agent, it has associations with a few conditions, namely, endocrine disorders, mitochondrial abnormalities, dermatological abnormalities, and infectious diseases. As the clinical features of this syndrome overlap with other conditions, many differential diagnoses should be ruled out to arrive at the final diagnosis of this syndrome [8].

The differential diagnoses include the conditions that cause intracranial calcifications, such as hyperparathyroidism, pseudo-hypoparathyroidism, tuberous sclerosis, toxoplasmosis, and syphilis, and inflammatory conditions like systemic lupus erythematosus.

Fahr's syndrome associated with hypoparathyroidism is rare and manifests with seizures and diffuse calcifications in the brain vessels and nasal ganglia [9]. This syndrome is also associated with hypocalcemic syndromes, which present with a loss of consciousness and convulsions [10]. Baseline biochemical investigations may be done to rule out other possible diagnoses. Molecular genetic testing can be done if there is a strong history of autosomal dominant inheritance [11].

The management of Fahr’s patients aims at symptomatic support, as there is no standard regime for the complete cure of the disease. Pharmacological treatment should be used to improve the condition of the patient. Antiepileptics are used for seizures and antipsychotics for depression and mental disorders. Seizures and movement disorders that are associated with parathyroid dysfunction can be resolved with the correction of phosphate and calcium levels by prescribing them alpha hydroxyvitamin D3 and corticosteroids. Clonazepam and atypical antipsychotics are also effective in treating patients with Fahr’s syndrome.

Prevention is always better than cure. So the correct time for preventing the risk is before pregnancy. Suspected, at-risk individuals should volunteer themselves for testing and genetic counseling to make their decisions on conception and family planning. With a positive test result, individuals must be prepared for periodic treatment and follow-up.

## Conclusions

The symptoms of Fahr’s syndrome are mainly due to bilateral calcifications in the brain, which cause neurodegenerative problems in these patients. Although this disorder has associations with a variety of other diseases, the specific etiologic agent has not been identified yet. Hence, more research has to be carried out on understanding the genetic disorder involved in the disease, and new treatment strategies should be formulated for preventing and curing the disease with safe and effective medical treatments.

## References

[1] Saleem S, Aslam HM, Anwar M, Anwar S, Saleem M, Saleem A, Rehmani MAK (2013). Fahr’s syndrome: literature review of current evidence. Orphanet J Rare Dis.

[2] Manyam BV, Walters AS, Narla KR (2001). Bilateral striopalliododentate calcinosis: clinical characteristics of patients seen in a registry. Mov Disord.

[3] Moskowitz MA, Winickoff RN, Heinz ER (1971). Familial calcification of basal ganglions: a metabolic and genetic study. N Engl J Med.

[4] Ellie E, Julien J, Ferrer X (1989). Familial idiopathic striopallidodentate calcifications. Neurology.

[5] Manyan BV (2005). What is and what is not Fahr’s disease. Parkinsonism Relt Disord.

[6] Kobari M, Nogawa S, Sugimoto Y, Fukuuchi Y (1997). Familial idiopathic brain calcification with autosomal dominant inheritance. Neurology.

[7] Asokan M, D’Souza S, Jeganathan J, Pai S (2013). Fahr’s syndrome - an interesting case presentation. J Clin Diagn Res.

[8] Verulashvili IV, Glonti L, Miminoshvili DK, Maniia MN, Mdivani KS (2006). Basal ganglia calcification: clinical manifestations and diagnostic evaluation [Article in Russian]. Georgian Med News.

[9] Swami A, Kar G (2011). Intracranial haemorrhage revealing pseudohypoparathyroidism as a cause of Fahr syndrome. Case Rep Neurol Med.

[10] Arranz PM, Ergueta MP, Gonzalez SE, Maranon CA (1992). Fahr’s disease and hypocalcemic syndromes. Presentation of a clinical case [Article in Spanish]. An Med Interna.

[11] Ooi HW, Er C, Hussain I, Kuthiah N (2019). Bilateral basal ganglia calcification: Fahr's disease. Cureus.

